# Porcine Reproductive and Respiratory Syndrome Virus NSP8 Suppresses NF-κB Signaling by Hijacking Host UBE2K and IKKα

**DOI:** 10.3390/v18050567

**Published:** 2026-05-18

**Authors:** Da Liu, Yan Yan, Xuezhen Fu, Linglong Qin, Jiayu Ma, Hui Zhou, Shiping Sun, Haimin Li, Weiren Dong, Jiyong Zhou

**Affiliations:** 1 MOA Key Laboratory of Animal Virology, Provincial Engineering Research Center of Animal Biological Products, Zhejiang University Center for Veterinary Sciences, Hangzhou 310058, China; 15376599380@163.com (D.L.); yyan1512@163.com (Y.Y.); 22317126@zju.edu.cn (X.F.); 15655375830@163.com (L.Q.); majy1000@163.com (J.M.); 12317041@zju.edu.cn (H.Z.); spsun@zju.edu.cn (S.S.); 11717037@zju.edu.cn (H.L.); 2 State Key Laboratory for Diagnosis and Treatment of Severe Infectious Diseases, First Affiliated Hospital, Zhejiang University, Hangzhou 310003, China

**Keywords:** UBE2K, PRRSV, NSP8, IKKα, NF-κB, ubiquitination

## Abstract

The Porcine Reproductive and Respiratory Syndrome Virus (PRRSV) has evolved sophisticated immune-evasion strategies to establish a productive infection in the host, primarily by counteracting the innate antiviral response. Here, we demonstrate for the first time that the PRRSV non-structural protein NSP8 suppresses NF-κB-dependent antiviral signalling by hijacking the host ubiquitin-conjugating enzyme UBE2K and inducing the degradation of IKKα, a pivotal kinase in the NF-κB pathway. PRRSV infection led to significant upregulation of host UBE2K, which in turn facilitated viral replication. Mechanistically, we found that NSP8 interacts directly with IKKα, triggering its degradation by the proteasome. Furthermore, we revealed that this process was facilitated by the host protein UBE2K, which acted as a crucial cofactor by directly interacting with NSP8 and thereby enhancing its activity against IKKα. This disruption blocked the activation of the NF-κB pathway and suppressed the expression of downstream antiviral factors, such as TNF-α, IL-6 and IFN-β, ultimately facilitating PRRSV replication. All of these findings showed that NSP8 is an important part of the process by which the host NF-κB pathway is blocked by viruses. This is a new way in which PRRSV avoids the immune system.

## 1. Introduction

Porcine Reproductive and Respiratory Syndrome (PRRS), a highly infectious and immunosuppressive disease caused by the porcine reproductive and respiratory syndrome virus (PRRSV), poses a grave threat to the global swine industry, resulting in considerable economic losses [1,2]. The virus is characterized by its rapid genetic variation and sophisticated immune evasion mechanisms [3], which not only contribute to the limited efficacy of current vaccines but also render disease prevention and control exceptionally challenging [4].

Eight structural proteins and at least sixteen non-structural proteins (NSPs) are encoded by PRRSV [5]. Structural proteins are mainly involved in virion assembly, budding, receptor recognition, and cellular entry [6,7]. NSPs are very important in putting together the viral replication–transcription complex (RTC) and in avoiding the immune system [8,9]. Accumulating evidence indicates that PRRSV employs multiple strategies to evade host immunity, which can be broadly categorized into five distinct mechanisms. First, antigenic variation and glycan shielding contribute to humoral immune evasion. For instance, GP5, a representative structural protein, harbors critical neutralizing epitopes within its ectodomain that are masked by adjacent N-glycosylation sites, thereby impairing the recognition by neutralizing antibody [10]. Second, PRRSV suppresses pattern recognition receptor signaling and the induction of type I interferon (IFN). For example, NSP11 downregulates RIG-I and MAVS, while NSP4 cleaves NEMO, collectively attenuating type I IFN production and early antiviral responses [11]. Third, PRRSV blocks IFN signaling and the effector functions of IFN-stimulated genes (ISGs). NSP1β induces degradation of KPNA1, thereby preventing nuclear translocation of ISGF3 and inhibiting JAK-STAT signaling pathway, which ultimately suppresses ISG expression [12]. Fourth, PRRSV hijacks ubiquitination-related degradation pathways to subvert innate immunity. NSP1α promotes the proteasomal degradation of TRIM25, whereas NSP2 facilitates the autophagic degradation of SH3KBP1, leading to disruption of K63-linked ubiquitination of RIG-I and subsequent impairment of innate immune signaling [13,14]. Fifth, PRRSV weakens antigen presentation and establishes an immunosuppressive microenvironment. The nucleocapsid (N) protein, for instance, promotes IL-10 production and induces regulatory T-cell while interfering with antigen presentation, thereby contributing to immune suppression [15]. Nevertheless, whether additional NSPs participate in host immune evasion remains largely unknown.

Ubiquitination has attracted considerable attention due to its vital role in controlling the innate immune response of the host, interferon signalling and viral replication during infection [16]. As an essential post-translational modification, ubiquitination is typically accomplished through a sequential three-enzyme cascade involving ubiquitin-activating enzymes (E1s), ubiquitin-conjugating enzymes (E2s), and ubiquitin ligases (E3s) [17]. In this cascade, E1s activate ubiquitin in an ATP-dependent manner to form an E1~Ub thioester intermediate. Ubiquitin is subsequently transferred to E2s via trans-thiolation to generate an E2~Ub conjugate. E3 ligases then recognise specific substrates. They also recruit E2~Ub. This catalyses the formation of an isopeptide bond between the C-terminus of ubiquitin and a lysine residue on the substrate protein. This results in monoubiquitination or polyubiquitin chain conjugation [18,19]. Ubiquitination has two sides to it in the way viruses and the body interact. On the one hand, the host employs the ubiquitin–proteasome system to degrade viral proteins or to activate antiviral signaling pathways [20]. On the other hand, viruses frequently hijack the host ubiquitination machinery to degrade key immune molecules, thereby antagonizing antiviral responses and establishing a cellular environment favorable for viral replication [21,22].

To date, studies investigating the role of ubiquitination in PRRSV infection have predominantly focused on host E3 ligases. For instance, RNF122 was shown to stabilize NSP4 and promote MDA5 degradation [23]; ASB8 stabilized NSP1α and facilitated the degradation of host IKKβ [24]; and TRIM28 inhibited the ubiquitin-mediated degradation of GP4, thereby enhancing viral replication [25]. By contrast, the involvement of host E2 enzymes in PRRSV infection remains poorly understood. Thus far, UBE2L6 is the only host E2 reported to be hijacked by NSP5 to promote the degradation of RIG-I and MDA5 and to suppress ISGylation, ultimately facilitating viral replication [26]. The precise roles of other host E2 enzymes during PRRSV infection, as well as the underlying molecular mechanisms, remain largely unexplored.

To establish persistent infection within the host, PRRSV has evolved sophisticated and diverse strategies to evade innate immune responses [11]. As a pivotal regulator of antiviral immunity, the NF-κB signaling pathway constitutes a significant target of viral antagonism [2]. IKKα is a catalytic subunit of the IKK complex and, although it has traditionally been linked to non-canonical NF-κB signaling, recent evidence indicates that it also makes an important contribution to canonical NF-κB activation in specific cellular contexts [27]. NF-κB is a central transcription factor family in innate immunity that coordinates inflammatory and antiviral gene programs downstream of pattern-recognition signaling [28]. Upon viral infection, innate immune signaling pathways frequently bifurcate toward NF-κB and IRF activation, and these transcriptional programs cooperate to induce antiviral mediators, including IFN-β. IFN-β is a key type I interferon that is rapidly induced after viral sensing and establishes an antiviral state by inducing interferon-stimulated genes in infected and neighboring cells [29]. In addition, TNF-α and IL-6 are major inflammatory cytokines downstream of NF-κB that participate in the early innate immune response and shape the antiviral inflammatory milieu [30]. Therefore, viral targeting of the IKKα/NF-κB axis represents an effective strategy to dampen host innate immune signaling and facilitate viral replication [31]. It has been shown by earlier research that PRRSV-encoded proteins disrupt NF-κB activation in several different ways. For instance, the linear ubiquitin chain assembly complex (LUBAC) is targeted by NSP1α to suppress NF-κB signaling [32]. NSP4, a 3C-like protease, cleaves IKKβ to weaken IKK complex-mediated signaling [33]. Additionally, the OTU domains of NSP2 and NSP11 have been shown to inhibit NF-κB activation via their deubiquiting activities, thereby promoting viral replication [34,35]. Collectively, these findings indicate that PRRSV NSPs antagonize NF-κB signaling at multiple levels. Despite these advances, the roles of host E2 ubiquitin-conjugating enzymes in PRRSV replication, as well as their functional interplay with viral NSPs, remain poorly understood. The present study was therefore designed to investigate the involvement of E2 enzymes in PRRSV replication, to identify NSPs capable of interacting with host E2s, and to explore the mechanistic link between E2s and NSPs in regulating the ubiquitination-dependent degradation of key kinases in the NF-κB pathway. This study provides new information about the complicated relationship between PRRSV and the host. It also offers a theoretical foundation and possible molecular targets for the development of new antiviral strategies.

## 2. Materials and Methods

### 2.1. Cells and Virus

MARC-145 and HEK-293T cells were maintained in our laboratory. Cells were cultured in Dulbecco’s Modified Eagle Medium (DMEM) (Gibco, Grand Island, NY, USA) supplemented with 10% fetal bovine serum (FBS) (ExCell, Suzhou, China) at 37 °C in a humidified incubator with 5% CO_2_. The PRRSV ZJU06C strain was propagated in MARC-145 cells.

### 2.2. Reagents and Antibodies

A Plasmid Miniprep Kit (DP103-02) was purchased from Tiangen Biotech (Beijing, China). Regular agarose (BY-R0100) was purchased from Shanghai Baijing Biotechnology (Shanghai, China). qPCR 96-well plates and sealing films were purchased from Hangzhou Weituo Biotechnology (Hangzhou, China). Antibodies against GAPDH (ET1601-4), Histone (HA600047), Myc tag (R1208-1), Flag tag (0912-1), GFP tag (ET1607-31), GST tag (ET1611-47), and 6 × His tag (PSH07-10) were purchased from HUABIO (Hangzhou, China). Antibodies against IKKα (Ab109721) and p65 (Ab117947) were purchased from Aladdin (Shanghai, China). An antibody against ubiquitin (A19686) was purchased from ABclonal (Wuhan, China). Mouse monoclonal antibody against PRRSV N and mouse polyclonal antibody against UBE2K were prepared and stored in our laboratory. MG132 (HY-13259) and CQ (HY-17589A) were purchased from MedChemExpress (Monmouth Junction, NJ, USA). NP-40 lysis buffer (P0013F), RIPA lysis buffer (P0013C), and the Dual-Luciferase Reporter Assay Kit (RG005) were purchased from Beyotime Biotechnology (Shanghai, China). A 4× protein loading buffer (P1016) was purchased from Solarbio (Beijing, China). A protein marker (26619), reverse transcription kit (K16225), and DNA marker (GeneRuler 100 bp Plus DNA Ladder, 91270326) were purchased from Thermo Fisher Scientific (Waltham, MA, USA). Anti-Flag immuno-agarose beads (A2220) and GST resin (L00206-50) were purchased from Hangzhou Youke Biotechnology (Hangzhou, China). Pierce™ Anti-c-Myc Magnetic Beads (88843) were purchased from Invitrogen Trading (Shanghai) Co., Ltd. (Shanghai, China). 2 × Taq Plus Master Mix II (Dye Plus) (P213-03), RNA isolater Total RNA Extraction Reagent (R401-01), high-fidelity DNA polymerase (P510-01), and Taq Pro Universal SYBR qPCR Master Mix (Q712-02) were purchased from Vazyme (Nanjing, China). JET transfection reagent (PT-114-15) was purchased from Polyplus (Illkirch-Graffenstaden, France). HRP-conjugated goat anti-rabbit and goat anti-mouse secondary antibodies were purchased from KPL (Gaithersburg, MD, USA). Chemiluminescent substrate was purchased from Biobest (Rochefort, Belgium).

### 2.3. Primer Design and Plasmid Construction

Plasmids encoding ubiquitin-conjugating enzymes (UBE2s) were preserved in our laboratory. Open reading frames (ORFs) of UBE2K, IKKα, and PRRSV genes were cloned into pCMV-Flag-N and pCMV-Myc-N to generate pCMV-Flag/Myc-UBE2K and pCMV-Myc/Flag-IKKα, and the corresponding PRRSV expression plasmids with Flag/Myc tag. NSP6 and NSP8 were cloned into pCMV-Flag-GST-N and pEGFP-C3 to generate pCMV-Flag-GST-NSP6, pCMV-Flag-GST-NSP8, and pEGFP-C3-NSP8. Primers used for qRT-PCR and for generation of knockout cell lines were designed and synthesized by Zhejiang Shangya Biotechnology (Hangzhou, China). Primer sequences were listed in Table 1.

### 2.4. Virus Infection Assay

MARC-145 cells were seeded in 6-well or 12-well plates. When cells reached 70–80% confluence, they were infected with PRRSV ZJU06C at an MOI of 1 for 2 h. The inoculum was removed, cells were washed 2–3 times with sterile PBS, and fresh DMEM containing 2% FBS was added. Samples were collected at 12, 24, or 36 h post infection (hpi).

### 2.5. RNA Extraction and Quantitative Real-Time PCR (qRT-PCR)

Total RNA was extracted using TRIzol reagent (Vazyme, Nanjing, China), and the reverse transcription was performed using a PrimeScript RT kit. The qRT-PCR assay was performed using Taq Pro Universal SYBR qPCR Master Mix (Vazyme, Nanjing, China) on a real-time PCR system. Relative gene expression was calculated using the 2^−ΔΔCt^ method.

### 2.6. Western Blotting

Cell lysis was carried out in RIPA buffer and the samples were combined with 4× protein loading buffer. Samples were subjected to boiling at 100 °C for a period of 15 min. Proteins were separated by SDS-PAGE. They were transferred onto 0.45 μm nitrocellulose membranes. The membranes were blocked with 5% non-fat milk in PBS for one hour at room temperature. They were then washed three times with PBST. After this, the membranes were incubated with primary antibodies at 4 °C overnight. After washing five times with PBST, the membranes were incubated with HRP-conjugated secondary antibodies for 2 h at room temperature. Following a wash with PBST, the signals were analysed by enhanced chemiluminescence.

### 2.7. Co-Immunoprecipitation (Co-IP)

The cells were lysed in NP-40 buffer containing PMSF at 4 °C for 4 h, after which they were centrifuged at 12,000× *g* for 10 min at 4 °C. A portion of the supernatant was retained as the input sample. The remaining lysate was pre-cleared with protein A/G agarose at 4 °C for 1 h, and then incubated with the corresponding antibody for the bait protein at 4 °C for 4 h. Approximately 60 μL of protein A/G-plus agarose was then added, and the mixture was incubated at 4 °C for a further 4 h to capture the immune complexes. The beads were then washed five times with PBS and eluted in strong lysis buffer and protein loading buffer by boiling for 10 min. The proteins were analysed by Western blotting using the indicated antibodies.

### 2.8. Denaturing Ubiquitination IP Assay

Cells were cultured for 36 h and then treated with MG132 at a final concentration of 10 μM for 6 h before harvest. Cell samples were lysed in stringent RIPA lysis buffer containing PMSF and 0.1% SDS at 4 °C for 4 h. The remaining procedures were performed as described for the Co-IP assay.

### 2.9. GST Pull-Down Assay

Purified GST or GST-NSP8 protein (20 μg) was incubated with an equal amount of purified His-UBE2K protein at 4 °C for 2–4 h. GST resin (50 μL) was added and incubated at 4 °C for another 2–4 h. After washing 5–6 times, bound proteins were eluted with strong RIPA buffer and protein loading buffer by boiling for 10 min and analyzed by Western blotting.

### 2.10. Virus Titration (TCID_50_) Assay

The fluid that had been extracted from the PRRSV-infected cells was then collected and serially diluted 10-fold in DMEM that contained 2% FBS. The MARC-145 cells, which had been seeded in 96-well plates, were treated with dilutions, with eight replicate wells per dilution. After 72 h’ incubation, CPE were recorded and viral titers were calculated using the Reed–Muench method.

### 2.11. Dual-Luciferase Reporter Assay

HEK-293T cells were seeded in 24-well plates. When the cells were almost full, the NF-κB-Luc reporter plasmid and pRL-TK were added together with the expression plasmids that were shown to be needed. At 24 h post-transfection, the cells were treated with the indicated stimuli. Luciferase activity was then measured using a Dual-Luciferase Reporter Assay Kit.

### 2.12. Generation of UBE2K-Knockout MARC-145 Cell Line

The design of sgRNAs targeting UBE2K was followed by the insertion of annealed oligonucleotides into the PCDH-CMV-MCS-EF1-Puro vector. The resulting constructs were co-transfected into HEK-293T cells. The transfection was with lentiviral packaging plasmids (psPAX2.0 and pMD). The ratio of the plasmids was 4:3:1. The culture supernatant was harvested after 48 h and used to infect MARC-145 cells. The cells were passaged and selected with 10 μg/mL puromycin 36 h after infection. The expression of UBE2K in the resulting MARC-145 cell line was analysed by Western blotting.

### 2.13. Statistical Analysis

Each experiment was repeated at least three times. Data are presented as the mean ± standard deviation (SD). Statistical analyses were performed using GraphPad Prism 9. Statistically significant differences were considered to be those with a *p*-value less than 0.05 (* *p* < 0.05, ** *p* < 0.01, *** *p* < 0.001, **** *p* < 0.0001; ns, *p* > 0.05).

## 3. Results

### 3.1. PRRSV Infection Upregulated UBE2K Expression and UBE2K Facilitates Viral Replication

**Figure 1:**
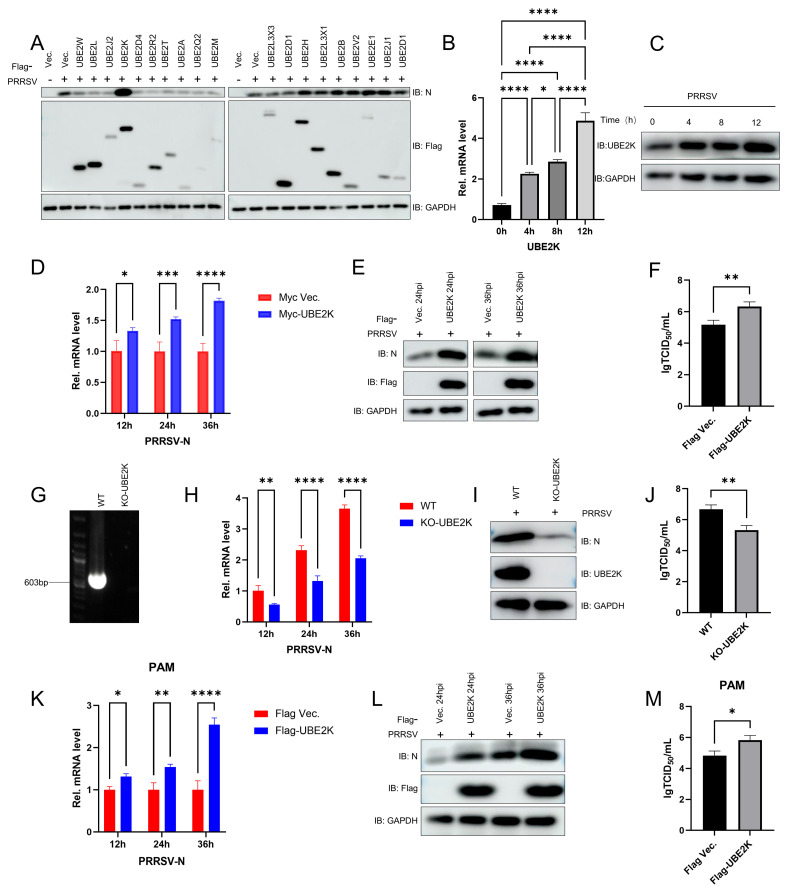
UBE2K facilitated the PRRSV replication. (**A**) Marc-145 cells were transfected with 2 μg of empty vector or one of 19 plasmids expressing Flag-tagged UBE2 proteins. At 24 h post-transfection, the cells were infected with PRRSV at an MOI of 1 and cultured for an additional 24 h, followed by detection of N, Flag, and GAPDH protein expression by Western blotting. (**B**,**C**) Marc-145 cells were infected with PRRSV at an MOI of 1, and the relative mRNA level of UBE2K was measured by RT-qPCR at 0, 4, 8, and 12 h post-infection, while the protein levels of UBE2K and GAPDH were analyzed by Western blotting. (**D**,**E**) Marc-145 cells were transfected with 2 μg of empty vector or a plasmid expressing Flag/Myc-UBE2K, and 24 h later were infected with PRRSV at an MOI of 1. The relative mRNA level of N was determined by RT-qPCR at 12, 24, and 36 h post-infection, and the protein levels of N, Flag, and GAPDH were analyzed by Western blotting at 24 and 36 h post-infection. (**F**) In parallel, Marc-145 cells transfected with empty vector or Flag-UBE2K were infected with PRRSV at an MOI of 1 for 24 h, subjected to three freeze–thaw cycles, and the resulting supernatants were used to infect fresh Marc-145 cells; cytopathic effects (CPE) were observed after a further 72 h of incubation. (**G**) The UBE2K-knockout (KO) Marc-145 cell line was verified by PCR. (**H**,**I**) Wild-type and UBE2K-KO Marc-145 cells were infected with PRRSV at an MOI of 1, and the relative mRNA level of N was measured by RT-qPCR at 12, 24, and 36 h post-infection, whereas the protein levels of N, UBE2K, and GAPDH were analyzed by Western blotting at 24 h post-infection. (**J**) Wild-type and UBE2K-KO Marc-145 cells were also infected with serially diluted PRRSV, and CPE was examined after 72 h of incubation. (**K**,**L**) PAM cells were transfected with 2 μg of empty vector or a plasmid expressing Flag-UBE2K, and 36 h later were infected with PRRSV at an MOI of 0.5. The relative mRNA level of N was determined by RT-qPCR at 12, 24, and 36 h post-infection, and the protein levels of N, Flag, and GAPDH were analyzed by Western blotting at 24 and 36 h post-infection. (**M**) PAM cells transfected with empty vector or Flag-UBE2K were infected with PRRSV at an MOI of 0.5 for 36 h, subjected to three freeze–thaw cycles, and the resulting supernatants were used to infect fresh Marc-145 cells; cytopathic effects (CPE) were observed after a further 72 h of incubation. All experiments were performed with at least three independent replicates. Data are presented as mean ± SD. Differences were considered statistically significant at *p* < 0.05 (* *p* < 0.05, ** *p* < 0.01, *** *p* < 0.001, **** *p* < 0.0001).

To investigate whether host E2 ubiquitin-conjugating enzymes play a role in the PRRSV replication, we screened a panel of 19 E2 enzymes for their functional role in protein expression. Only overexpression of UBE2K in MARC-145 cells markedly increased the expression of the viral N protein following PRRSV infection (Figure 1A). Conversely, PRRSV infection itself led to a time-dependent upregulation of both endogenous UBE2K mRNA and protein levels (Figure 1B,C). Overexpression of UBE2K boosted ORF7 transcription (Figure 1D), significantly enhanced N protein expression at 24 and 36 hpi (Figure 1E), and ultimately elevated viral titers (Figure 1F). UBE2K-knockout (KO) MARC-145 cells were generated using CRISPR/Cas9 technology, which resulted in an undetectable level of UBE2K protein (Figure 1G). In these KO cells, PRRSV ORF7 mRNA levels and N protein expression were significantly reduced upon infection (Figure 1H,I), and viral titers were markedly decreased (Figure 1J). To enhance the physiological relevance of the study, we also performed the experiments in PAM cells. The results showed that overexpression of UBE2K likewise significantly increased the transcription level of PRRSV ORF7, the expression level of the N protein, and viral titers (Figure 1K–M).

### 3.2. Screening of PRRSV Nonstructural Proteins Interacting with UBE2K

**Figure 2:**
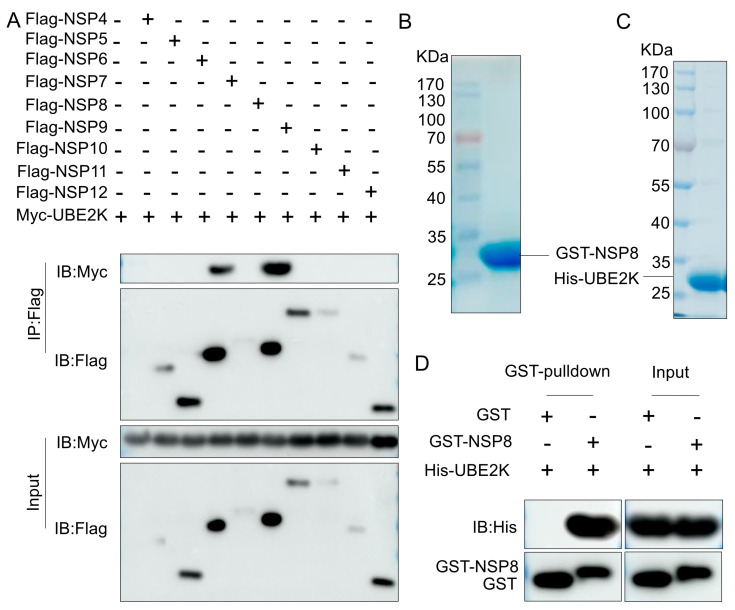
UBE2K interacted with PRRSV nonstructural protein NSP8. (**A**) HEK293T cells were co-transfected with 2 μg of empty vector or plasmids expressing Flag-tagged NSPs together with 2 μg of a plasmid expressing Myc-UBE2K. At 36 h post-transfection, the cells were harvested for Co-IP, and the expression of Myc and Flag proteins was analyzed by Western blotting. (**B**,**C**) Purified GST-NSP8 protein and His-UBE2K protein were subjected to SDS-PAGE followed by Coomassie brilliant blue staining. (**D**) Protein samples from the GST pull-down assay were analyzed by Western blotting for the expression of His and GST proteins.

To investigate whether viral proteins promote PRRSV replication through interaction with UBE2K following infection, we performed Co-IP screening of major PRRSV NSPs. Among the nine NSPs tested, NSP8 and NSP6 were found to interact with UBE2K, with NSP8 exhibiting a relatively stronger interaction signal (Figure 2A). Therefore, NSP8 was selected for subsequent analyses. To assess whether NSP8 directly interacts with UBE2K, GST-tagged NSP8 and His-tagged UBE2K were expressed and purified (Figure 2B,C). An in vitro GST pull-down assay confirmed a direct interaction between NSP8 and UBE2K (Figure 2D).

### 3.3. NSP8 Interacted with IKKα and Induced Proteasome-Dependent Degradation

**Figure 3:**
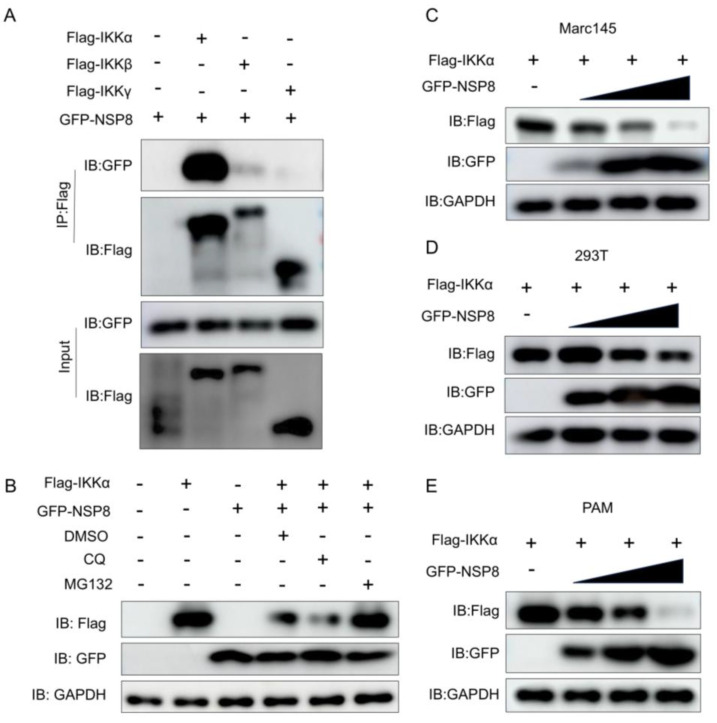
NSP8 interacted with IKKα and induced proteasome-dependent degradation of IKKα. (**A**) HEK293T cells were co-transfected with 2 μg of empty vector or plasmids expressing Flag-tagged IKKs together with 2 μg of a plasmid expressing GFP-NSP8. At 36 h post-transfection, the cells were harvested for Co-IP, and the expression of GFP and Flag proteins was analyzed by Western blotting. (**B**) Marc-145 cells were co-transfected with 1 μg of empty vector or a plasmid expressing Flag-IKKα together with 1 μg of a plasmid expressing GFP-NSP8. At 24 h post-transfection, the cells were treated with MG132 (final concentration, 10 μM) or CQ (final concentration, 50 μM) for 12 h, followed by Western blot analysis of Flag, GFP, and GAPDH protein levels. (**C**–**E**) HEK293T, Marc-145 and PAM cells were co-transfected with 1 μg of a plasmid expressing Flag-IKKα together with empty vector or 0.1, 0.5, or 1 μg of a plasmid expressing GFP-NSP8. The total amount of plasmid in each group was kept constant at 2 μg by supplementing with empty vector as needed. At 24 h post-transfection, the protein levels of Flag, GFP, and GAPDH were analyzed by Western blotting.

Viral proteins can disrupt host innate immunity by interacting with different host proteins. Therefore, we selected several key signaling nodes, including MDA5, MAVS, TBK1, and the IKK complex, for investigation. The results revealed that NSP8 strongly interacted with IKKα, a critical kinase in the NF-κB pathway, whereas only a relative weak interaction was detected with IKKβ and no binding was observed with IKKγ (Figure 3A). Subsequently, we conducted further investigations focusing on IKKα. Functional studies demonstrated that overexpression of NSP8 in HEK-293T, MARC-145 and PAM cells led to a dose-dependent reduction in IKKα protein levels (Figure 3C–E). Further mechanistic dissection showed that treatment with the proteasome inhibitor MG132, but not the lysosomal inhibitor CQ, rescued IKKα protein abundance in NSP8-expressing cells (Figure 3B). These findings indicated that NSP8 promoted proteasome-dependent degradation of IKKα.

### 3.4. UBE2K Enhanced NSP8-Mediated Ubiquitination and Degradation of IKKα

**Figure 4:**
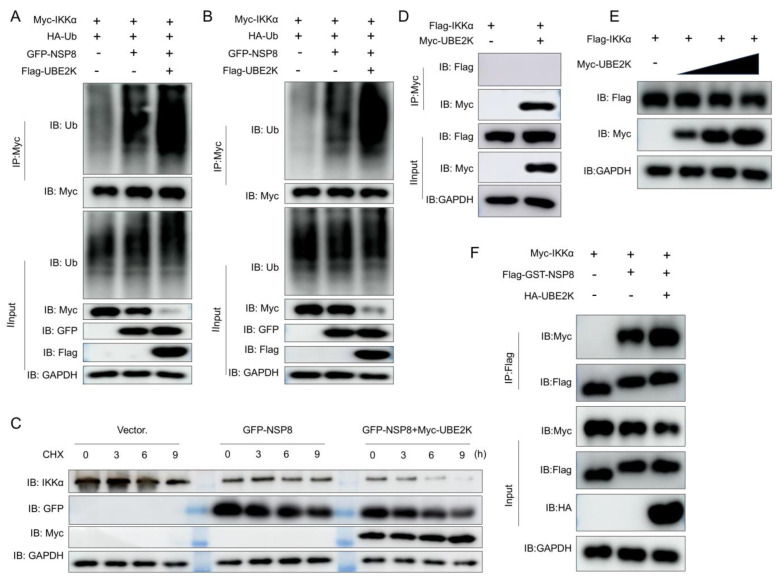
UBE2K enhanced NSP8-mediated ubiquitination and degradation of IKKα. (**A**) HEK293T cells were co-transfected with 1 μg of a plasmid expressing Myc-IKKα, 1.5 μg of a plasmid expressing HA-Ub, and 1 μg of empty vector, a plasmid expressing GFP-NSP8, or a plasmid expressing Flag-UBE2K. At 36 h post-transfection, the cells were harvested for Co-IP, and the protein levels of ubiquitin, Myc, Flag, GFP, and GAPDH were analyzed by Western blotting. (**B**) The procedure was essentially the same as in A, except that the cells were lysed with strong RIPA lysis buffer containing SDS. (**C**) Marc-145 cells were transfected with 1 μg of empty vector or a plasmid expressing GFP-NSP8 alone, or co-transfected with 1 μg of a plasmid expressing GFP-NSP8 and 1 μg of a plasmid expressing Myc-UBE2K. At 24 h post-transfection, the cells were treated with CHX (100 μg/mL) and harvested at 0, 3, 6, and 9 h, followed by Western blot analysis of IKKα, GFP, Myc, and GAPDH protein levels. (**D**) HEK293T cells were co-transfected with 2 μg of empty vector or a plasmid expressing Myc-UBE2K together with 2 μg of a plasmid expressing Flag-IKKα. At 36 h post-transfection, the cells were harvested for Co-IP, and the expression of Myc, Flag, and GAPDH proteins was analyzed by Western blotting. (**E**) Marc-145 cells were co-transfected with 1 μg of a plasmid expressing Flag-IKKα together with empty vector or 0.1, 0.5, or 1 μg of a plasmid expressing Myc-UBE2K. The total amount of plasmid in each group was kept constant at 2 μg by supplementing with empty vector as needed. At 24 h post-transfection, the protein levels of Flag, Myc, and GAPDH were analyzed by Western blotting. (**F**) HEK293T cells were co-transfected with 2 μg of Flag-GST vector or a plasmid expressing Flag-GST-NSP8 together with 2 μg of a plasmid expressing Myc-IKKα, one group was additionally transfected with 1 μg of a plasmid expressing HA-UBE2K. At 36 h post-transfection, the cells were harvested for Co-IP, and the expression of Myc, Flag, HA and GAPDH proteins was analyzed by Western blotting.

The observation that UBE2K directly interacted with NSP8 prompted an investigation into whether UBE2K participated in NSP8-mediated ubiquitination and degradation of IKKα. Co-expression of UBE2K significantly enhanced NSP8-induced ubiquitination of IKKα (Figure 4A,B) and accelerated the degradation of IKKα (Figure 4C). Analysis of the interaction network revealed that NSP8 interacted with both UBE2K and IKKα, whereas no binding between UBE2K and IKKα was detectable (Figure 4D). Consistent with this observation, overexpression of UBE2K alone was insufficient to drive efficient and reproducible IKKα degradation (Figure 4E). However, overexpression of UBE2K enhanced the interaction between NSP8 and IKKα (Figure 4F). The data demonstrate that NSP8 promoted ubiquitin degradation of IKKα by interaction with UBE2K.

### 3.5. NSP8 and UBE2K Cooperatively Inhibited IKKα-Mediated NF-κB Signaling

**Figure 5:**
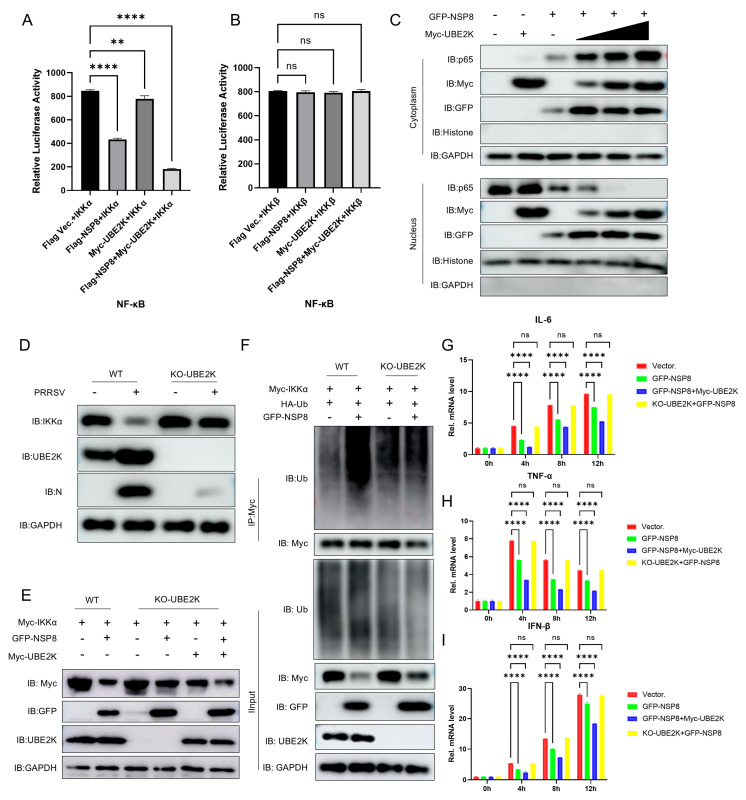
UBE2K cooperated with NSP8 to inhibit the IKKα/NF-κB signaling pathway. (**A**,**B**) HEK293T cells were co-transfected with 500 ng of an NF-κB luciferase reporter plasmid, 25 ng of pRL-TK, and 500 ng of empty vector, a plasmid expressing Myc-UBE2K, a plasmid expressing GFP-NSP8, or both, together with 500 ng of a plasmid expressing Flag-IKKα or Flag-IKKβ as indicated. At 36 h post-transfection, the cells were harvested for luciferase activity assay. (**C**) Marc-145 cells were transfected with 1 μg of empty vector, a plasmid expressing GFP-NSP8, or a plasmid expressing Myc-UBE2K alone, or co-transfected with 1 μg of a plasmid expressing GFP-NSP8 together with empty vector or 0.1, 0.5, or 1 μg of a plasmid expressing Myc-UBE2K. The total amount of plasmid in each group was kept constant at 2 μg by supplementing with empty vector as needed. Cell lysates were subjected to nuclear and cytoplasmic fractionation, and the protein levels of p65, Myc, GFP, Histone, and GAPDH were analyzed by Western blotting. (**D**) Wild-type and UBE2K-knockout Marc-145 cells were infected with PRRSV at an MOI of 1. At 24 h post-infection, the cells were harvested and the protein levels of IKKα, UBE2K, N, and GAPDH were analyzed by Western blotting. (**E**) Wild-type and UBE2K-knockout Marc-145 cells were co-transfected with 1 μg of a plasmid expressing Myc-IKKα together with 1 μg of empty vector or a plasmid expressing GFP-NSP8. In parallel, UBE2K-knockout cells were reconstituted with 1 μg of a plasmid expressing Myc-UBE2K. At 24 h post-transfection, the cells were harvested and the protein levels of Myc, GFP, UBE2K, and GAPDH were analyzed by Western blotting. (**F**) Wild-type and UBE2K-knockout Marc-145 cells were co-transfected with 1 μg of a plasmid expressing Myc-IKKα, 1.5 μg of a plasmid expressing HA-Ub, and 1 μg of empty vector or a plasmid expressing GFP-NSP8. At 36 h post-transfection, the cells were harvested for Co-IP, and the protein levels of ubiquitin, Myc, GFP, UBE2K, and GAPDH were analyzed by Western blotting. (**G**–**I**) Wild-type Marc-145 cells were transfected with 1 μg of empty vector or a plasmid expressing GFP-NSP8 alone, or co-transfected with 1 μg of a plasmid expressing GFP-NSP8 and 1 μg of a plasmid expressing Myc-UBE2K. In parallel, UBE2K-knockout Marc-145 cells were transfected with 1 μg of a plasmid expressing GFP-NSP8. At 24 h post-transfection, the cells were treated with poly(I:C) at 1 μg/mL and harvested at 0, 4, 8, and 12 h. The relative mRNA levels of IL-6, TNF-α, and IFN-β were measured by RT-qPCR. All experiments were performed with at least three independent replicates. Data are presented as mean ± SD. Differences were considered statistically significant at *p* < 0.05 (** *p* < 0.01, **** *p* < 0.0001; ns, *p* > 0.05).

To determine whether UBE2K and NSP8 act synergistically to suppress NF-κB signaling, an NF-κB luciferase reporter assay was employed. Overexpression of NSP8 inhibited IKKα-mediated NF-κB transcriptional activity, an effect that was further potentiated by UBE2K co-expression. In contrast, overexpression of NSP8 and/or UBE2K did not affect IKKβ-mediated NF-κB activity (Figure 5A,B). Mechanistically, nuclear-cytoplasmic fractionation revealed that co-expression of UBE2K and NSP8 markedly reduced the nuclear accumulation of p65 (Figure 5C). To assess the dependency of NSP8 function on UBE2K, we generated UBE2K-knockout (KO) MARC-145 cells. Upon PRRSV infection, IKKα stability was significantly enhanced in UBE2K KO cells compared to wild-type controls (Figure 5D). Furthermore, NSP8-induced IKKα degradation and ubiquitination were severely impaired in the absence of UBE2K, which effects were fully restored upon UBE2K reconstitution (Figure 5E,F). Consistent with these observations, qRT-PCR analysis showed that NSP8 significantly inhibited poly(I:C)-induced transcription of TNF-α, IL-6, and IFN-β, with the most pronounced inhibition observed when UBE2K and NSP8 were co-expressed. In UBE2K KO cells, the inhibitory effect of NSP8 on these NF-κB downstream genes was markedly attenuated (Figure 5G–I). Collectively, these results indicated that NSP8-mediated degradation of IKKα and subsequent suppression of the NF-κB pathway were highly dependent on its cooperative interaction with UBE2K.

## 4. Discussion

The persistent circulation of PRRSV is largely attributed to its ability to subvert host innate immunity [36,37]. This virus establishes long-term replication in vivo and induces immunosuppression, primarily through the systematic disruption of key innate immune hubs mediated by its nonstructural proteins (NSPs) encoded within the replicase region [38,39,40]. The NF-κB pathway, a master regulator of pro-inflammatory cytokines and antiviral genes, is preferentially targeted by PRRSV. Collectively, these findings illustrate a multifaceted strategy whereby PRRSV employs multiple NSPs to antagonize NF-κB signaling at various levels of the cascade.

In this study, PRRSV infection was found to upregulate UBE2K expression. Functional assays revealed that UBE2K overexpression enhanced viral RNA levels, N protein expression, and viral titers, whereas UBE2K knockout produced the opposite effects, suggesting that PRRSV exploits UBE2K to gain a replication advantage. Mechanistically, UBE2K was shown to directly interact with the viral NSP8. NSP8 in turn bound to the immune signaling molecule IKKα and induced its proteasome-dependent degradation, which could be abrogated by MG132. Co-expression of UBE2K with NSP8 further enhanced IKKα ubiquitination and accelerated its degradation. In addition to facilitating NSP8-mediated ubiquitination and degradation of IKKα, UBE2K may also influence the stability or expression of NSP8 itself. In the present study, we demonstrated that UBE2K directly interacted with NSP8 and enhanced the interaction between NSP8 and IKKα, thereby potentiating the inhibitory effect of NSP8 on the IKKα/NF-κB axis. However, whether UBE2K regulates the protein abundance, turnover, or intracellular stability of NSP8 was not systematically examined in this work. Given that ubiquitin-conjugating enzymes can modulate not only substrate degradation but also the stability and functional state of interacting proteins, it will be of interest in future studies to determine whether UBE2K affects NSP8 itself, thereby further strengthening PRRSV immune evasion and replication. Notably, UBE2K alone did not show detectable binding to IKKα or induce obvious IKKα degradation; instead, it appeared to function mainly by cooperating with NSP8 and enhancing the interaction between NSP8 and IKKα, thereby promoting IKKα ubiquitination and degradation. These observations supported a model in which NSP8 interacted with and degraded IKKα via the ubiquitin–proteasome pathway, an effect further potentiated by the interaction between NSP8 and UBE2K. Although further study is required to define whether UBE2K potentiates IKKα ubiquitination directly or indirectly, this model offers a mechanistic basis for understanding how PRRSV achieves concurrent degradation of a pivotal kinase and suppression of downstream antiviral transcription within a single complex. These findings revealed a previously unrecognized regulatory paradigm in which a viral protein bridged a host E2 enzyme and immune signaling components to subvert host defense.

NSP8, encoded by ORF1a, represents the N-terminal extension of the viral RNA-dependent RNA polymerase (RdRp; NSP9) and is essential for viral RNA synthesis [41]. During early infection, NSP8 localizes to discrete punctate foci in the peri-nuclear region8 and is considered a core component of RTC formation [42]. UBE2K (also known as E2-25K or HIP2) is an atypical ubiquitin-conjugating enzyme that has drawn attention because it can synthesize ubiquitin chains independently of E3 ligases [43,44]. Accumulating evidence suggests that UBE2K may be involved in the PRRSV life cycle [45,46,47], but its precise role and regulatory network during PRRSV infection remain unclear. Degradation of IKKα has been shown to impair NF-κB pathway activation [27,48]. Consistently, NSP8 was found to inhibit NF-κB activity and reduce nuclear translocation of p65, which was accompanied by decreased transcription of downstream genes such as TNF-α, IL-6, and IFN-β. Importantly, knockout of UBE2K attenuated the inhibitory effect of NSP8 on the IKKα/NF-κB axis and suppressed viral replication, thereby functionally linking the proposed molecular mechanism to the observed infection phenotypes. Together with previous reports demonstrating that multiple PRRSV NSPs targeted key components of NF-κB pathway, our findings suggested that PRRSV might achieve more robust immunosuppression by targeting different subunits of the IKK complex.

Although the present study revealed a previously unrecognized mechanism by which PRRSV NSP8 cooperates with host UBE2K to promote IKKα degradation and suppress NF-κB-dependent antiviral responses, several limitations should be noted. First, the proposed mechanism was mainly established on the basis of in vitro experiments in cultured cells, and its in vivo relevance during PRRSV infection remains to be determined. Future studies in pigs, such as studies focusing on knockdown or inhibition of UBE2K or modulation of NSP8 expression, followed by assessment of viral replication and host immune responses, will be necessary to further confirm the physiological significance of the UBE2K–NSP8–IKKα ternary complex in PRRSV infection.

**Figure 6:**
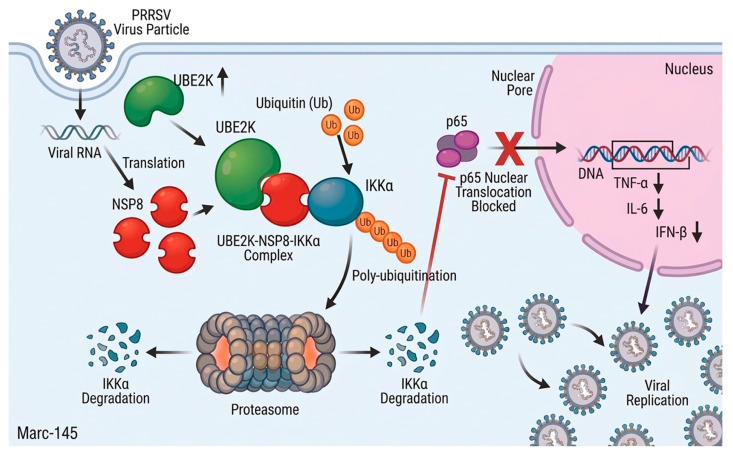
Proposed model. PRRSV infection upregulates UBE2K, which interacts with NSP8 to form a functional complex. NSP8 acts as a bridge to recruit UBE2K, thereby enhancing ubiquitination of IKKα and promoting its proteasome-dependent degradation. This suppresses NF-κB activation and ultimately facilitates PRRSV replication.

In summary, this study uncovered a potential molecular mechanism by which PRRSV modulated host innate immunity (Figure 6). The viral nonstructural protein NSP8 not only participated in viral replication but also exerted immunomodulatory activity by binding IKKα and promoted its proteasomal degradation, thereby suppressing NF-κB signaling and facilitating viral propagation. These findings identified, for the first time, NSP8 as a viral regulator targeting a core node of the NF-κB pathway, expanding the current understanding of how PRRSV nonstructural proteins contribute to immune evasion. Notably, this work revealed that replication-associated viral proteins can be “multi-functionalized” to achieve synergistic coupling between replication fitness and immune antagonism. Furthermore, the host E2 ubiquitin-conjugating enzyme UBE2K was identified as a critical proviral cofactor in this process, demonstrating that PRRSV hijacked the host ubiquitin system by employing a viral protein as a bridging adaptor to fine-tune IKKα stability. The characterization of the UBE2K–NSP8–IKKα ternary complex not only provided new insights into PRRSV–host interplay but also offered a new theoretical framework and potential targets for developing antiviral strategies aimed at disrupting virus–host protein interactions or host ubiquitination pathways.

## Figures and Tables

**Table 1:** Oligonucleotide primers used for gene cloning and plasmid construction in this study.

Primer Name	Restriction Site	Primer Sequence (5′–3′)	Purpose
NSP4-F	*EcoRI*	GCCATGGAGGCCCGAATGGGCGCTTTCAGAACTCAAAAG	Gene amplification
NSP4-R	*EcoRI*	CTCGGTCGACCGAATTCATTCCAGTTCGGGTTTGGC	
NSP5-F	*EcoRI*	GCCATGGAGGCCCGAATGGGAGGCCTTTCCACAGTTC	Gene amplification
NSP5-R	*EcoRI*	CTCGGTCGACCGAATTTCACTCGGCAAAGTATCGCAAG	
NSP6-F	*EcoRI*	GCGAATTCGGATGGGAAAGTTGAGGGAAGGGG	Gene amplification
NSP6-R	*KpnI*	GCGGTACCTCACTCATGACTCATCCCGCAG	
NSP7-F	*EcoRI*	GCCATGGAGGCCCGAATGTCGCTGACTGGTGCCCTCG	Gene amplification
NSP7-R	*EcoRI*	CTCGGTCGACCGAATTCATTCCCACTGAGCTCTTC	
NSP8-F	*EcoRI*	GCCATGGAGGCCCGAATGGCCGCCAAGCTTTCCGTGG	Gene amplification
NSP8-R	*KpnI*	GCGGTACCTCACTCATAGCAGTTTAAACACTGC	
NSP9-F	*EcoRI*	GCCATGGAGGCCCGAATGCCCTCTGGCTTTGAGTTG	Gene amplification
NSP9-R	*EcoRI*	CTCGGTCGACCGAATTTCACTCATGATTGGACCTGAG	
NSP10-F	*EcoRI*	GCCATGGAGGCCCGAATGGGGAAGAAGTCCAGAATG	Gene amplification
NSP10-R	*EcoRI*	CTCGGTCGACCGAATTCACAGGTCTGCGCAAATAGCG	
NSP11-F	*EcoRI*	GCCATGGAGGCCCGAATGGAAGGGTCGAGCTCCCCGC	Gene amplification
NSP11-R	*EcoRI*	CTCGGTCGACCGAATTCATTCAAGTTGAAAATAGGCC	
NSP12-F	*EcoRI*	GCCATGGAGGCCCGAATGGGCCGCCATTTTACCTGG	Gene amplification
NSP12-R	*EcoRI*	CTCGGTCGACCGAATTTCAATTCAGGCCTAAAGTTGG	
UBE2K-F	*EcoRI*	GCCATGGAGGCCCGAATGGCCAACATCGCG	Gene amplification
UBE2K-R	*EcoRI*	CTCGGTCGACCGAATTCAGTTACTCAGAAGCAATTCTG	
IKKα-F	*EcoRI*	GCCATGGAGGCCCGAATGGAGCGGCCCCCG	Gene amplification
IKKα-R	*EcoRI*	CTCGGTCGACCGAATTCATCCTGTTAACC	
Monkey-β-actin-F	/	GGCACCACACCTTCTACAAT	Quantitative detection
Monkey-β-actin-R	/	AACATGATCTGGGTCATCTTCTC	
PRRSV-ORF7-F	/	AAACCAGTCCAGAGGCAAGG	Quantitative detection
PRRSV-ORF7-R	/	GCAAACTAAACTCCACAGTGTAA	
Monkey-IL6-F	/	AGACAGCCACTCACCTCTTCAG	Quantitative detection
Monkey-IL6-R	/	TTCTGCCAGTGCCTCTTTGCTG	
Monkey-TNF-α-F	/	CTCTTCTGCCTGCTGCACTTTG	Quantitative detection
Monkey-TNF-α-R	/	ATGGGCTACAGGCTTGTCACTC	
Monkey-UBE2K-F	/	ATAGCAGGACCTCCAGACACAC	Quantitative detection
Monkey-UBE2K-R	/	CAAATAGCCCCTGTGACGGAAC	
Monkey-IFN-β-F	/	ATGACCAACAAGTGTCTCCTCC	Quantitative detection
Monkey-IFN-β-R	/	GCTCATGGAAAGAGCTGTAGTG	
sg-UBE2K-F	/	CACCGGGCCTTGTCTTCAAAATCATGGG	Gene knockout
sg-UBE2K-R	/	AAACCCCATGATTTTGAAGACAAGGCCC	

## Data Availability

The original contributions presented in the study are included in the article, and further inquiries can be directed to the corresponding authors.

## Abbreviations

The following abbreviations are used in this manuscript:

PRRSV	Porcine Reproductive and Respiratory Syndrome Virus
NSPs	Nonstructural proteins
ORFs	Open reading frames
IKK	IκB kinase
NF-κB	Nuclear factor kappa-light-chain-enhancer of activated B cells
p65	NF-κB p65 subunit (RelA)
TNF-α	Tumor necrosis factor alpha
IL-6	Interleukin 6
IFN-β	Interferon beta
UPS	Ubiquitin–proteasome system
CRISPR	Clustered regularly interspaced short palindromic repeats
Cas9	CRISPR-associated protein 9
sgRNA	single-guide RNA
MOI	multiplicity of infection
CPE	cytopathic effect
CQ	Chloroquine (autophagy inhibitor)
MG132	carbobenzoxy-Leu-Leu-leucinal (proteasome inhibitor)
